# Motivations Influencing Caffeine Consumption Behaviors among College Students in Korea: Associations with Sleep Quality

**DOI:** 10.3390/nu12040953

**Published:** 2020-03-30

**Authors:** Jinkyung Choi

**Affiliations:** Department of Foodservice Management, Woosong University, Daejeon 34606, Korea; choi3728@wsu.ac.kr; Tel.: +82-42-630-9253

**Keywords:** caffeine, caffeinated beverage, motivation, sleep quality, consumption

## Abstract

Caffeinated beverages are a part of daily life. Caffeinated beverages such as coffee, tea, energy drinks, and soft drinks are easy to purchase and are frequently consumed by young college students. Moreover, smoking influences the consumption of caffeinated beverages. The concentration of caffeine in these products is an attractive factor for individuals that desire the effects of caffeine; however, abusing such products may lead to poor sleep quality. The motivations that drive caffeinated beverage consumption were investigated in this study through a survey. Self-reported questionnaires were distributed on campus to students enrolled at a university in Korea. The motivations of the students for consuming each caffeinated beverage and their sleep quality were investigated. The results of exploratory factor analysis showed the motivations for caffeinated beverage consumption were alertness, taste, mood, socialization, health benefits, and habit. The motivations for consuming each caffeinated beverage product were different. For instance, coffee consumption was motivated by a desire for alertness (B = 0.107, SE = 0.049, t = 2.181, *p* < 0.05) and by habit (B = 0.345, SE = 0.046, t = 7.428, *p* < 0.001), whereas tea consumption was influenced by socialization (B = 0.142, SE = 0.060, t = 2.357, *p* < 0.05). Energy drink consumption was motivated by a desire for alertness (B = 0.100, SE = 0.034, t = 2.966, *p* < 0.01) and health benefits (B = 0.120, SE = 0.051, t = 2.345, *p* < 0.05), while the consumption of soft drinks was not motivated by any specific factors. Caffeinated beverage consumption did not show a significant relationship with sleep quality, although the general sleep quality of the respondents was poor. Smoking status showed significant differences in coffee and tea consumption as well as sleep quality. Smokers had a higher intake of coffee and a lower intake of tea than non-smokers. No interaction effect between smoking and coffee on sleep quality was found. Labeling detailing the amount of caffeine in products is necessary and a cautionary statement informing consumers that smoking cigarettes enhances the effects of caffeine should be included.

## 1. Introduction

Caffeine is part of our diet and is often consumed in different types of drinks and food such as coffee beans, tea leaves, cocoa beans, kola nuts, and other plants [1]. The average caffeine intake per day is approximately 128.8 mg in Korea [2] and between 210 and 238 mg for Americans through various types of food including caffeinate beverages [3]. Additionally, 89% of adults in the U.S. drink caffeinated beverages on a daily basis in the form of coffee (64%), soft drinks (18%), and tea (16%) [4], and Koreans have similar patterns [5]. Most consumers surveyed consumed caffeinated beverages, with 98% of all caffeinated beverages consumed as coffee, tea, soft drinks, and energy drinks, and coffee being the primary source of caffeine in the adult diet in many countries [4,5,6,7,8]. Coffee is the most common caffeinated beverage available to consumers [8] and is one of the most popular caffeinated beverages in Korea, with sales of 11 trillion and 7 hundred billion won in 2017, increasing from 3 trillion won in 2007 [9]. Caffeinated beverages have gained increasing popularity, especially among young generations, as lifestyles have become more Westernized. Food and beverage consumption trends indicate coffee is a preferred food rather than a functional food containing caffeine. However, most consumers are not aware of the amount of caffeine in their caffeinated drinks or its effects on them [8]. 

Factors associated with caffeine can be categorized as alertness, withdrawal symptoms, socializing, sensory effects, etc. Young generations, for example, most college students, consume caffeine to feel more awake, enjoy the taste, socialize, increase their physical energy [10], improve their mood, and alleviate stress [11]. Energy drinks are another type of caffeinated beverage popular among college students and the reasons for consuming energy drinks are frequently listed as increasing energy or counteracting insufficient sleep [10]. Among the many effects of caffeine, the enhancement of physical performance was reported in the early 20th century [12] and athletes frequently take advantage of caffeine as a stimulant [7,13]. The most common reason for taking caffeine is to feel more awake [11]. Caffeine is rapidly absorbed as quickly as 45 to 60 min [3] or 1–1.5 hours following ingestion and the absorbed caffeine is disseminated throughout the entire body, crossing the blood–brain barrier [1].

Nicotine can increase the speed of caffeine metabolism by almost 50% [14]. Previous literature has shown that smoking enhances caffeine metabolism, which also accelerates caffeine clearance [1]. The effects of caffeine on performance occur through adenosine receptors, which are related to the brain and are associated with sleep, arousal, and cognition [15,16]. In addition, the sensory elements of coffee have a societal impact, inducing mood changes [17]. According to an analysis of previous literature by O’Callahan [18], the positive and negative effects of caffeine coexist.

Although caffeine has some positive effects on general performance including physical and non-physical aspects, there are several negative side effects of taking caffeine. A study examined the effect of caffeine on 15,686 adolescents and found that students with high caffeine intake were more likely to feel worn-out compared to those who consumed less caffeine [19]. In addition, caffeinated energy drinks may be related to methylxanthine, which influences memory, anxiety, and sleep [7,10,20]. The consequences of taking caffeine can lead to insufficient sleep both in quality and quantity, which may result in the consumption of caffeine again to overcome the symptoms caused by the lack of sleep.

Laboratory studies have shown that a deficit in nocturnal sleep of as little as 90 min for just one night can lead to a one-third reduction in daytime objective alertness [21]. In another study investigating the effects of caffeine on sleep regulation, participants were administered 200 mg of caffeine early in the morning at 7 a.m. and monitored throughout the day. The caffeine significantly affected sleep efficiency as well as the total sleep time [22]. A mathematical model was developed to predict sleep reaction time according to the caffeine dose [23]. A study investigated the effectiveness of caffeinated beverages on excessive daytime sleepiness and sleep duration and found that participants who consumed more coffee had a shorter sleep duration (equal to or less than 6 hours) [24]. Despite the disadvantages of caffeinated beverages, consumers often ignore the negative effects of caffeine. These effects have inspired cautionary actions such as the recommendation of a maximum amount of caffeine consumption.

In Korea, the daily maximum intake of caffeine is 400 mg for adults and 300 mg for pregnant women [25]. The EU also recommends a maximum intake of 400 mg for adults and 200 mg for pregnant women [26]. Coffee contains the highest amount of caffeine (40–259 mg) among foods and beverages, followed by energy drinks (42–141 mg), teas (30–80 mg), soft drinks (36–71 mg), and chocolate products (12–25 mg) [27,28]. The EU requires a ‘high caffeine content’ label for products with caffeine in excess of 150 mg [28]. The US FDA does not require the amount of caffeine be displayed but caffeine must be listed as a product [29]. Recently, the Korean National Food and Drug Administration implemented a new requirement of labeling the total amount of caffeine in products including café-made coffee in order to prevent caffeine overdose [5]. 

Despite the popularity and the prevalence of consumption, only a few studies have investigated caffeinated beverage consumption behaviors [30]. With increasing concerns about the over-consumption of caffeine, it is essential to investigate the reasons why people consume caffeinated beverages. Understanding the motivation for consuming caffeinated beverages will lead to more effective regulations for labeling such information on products, increasing the success of advising consumers about high caffeine consumption. Consuming a product without knowing how much caffeine it contains may cause other problems as caffeine has both advantageous and disadvantageous effects. Hence, the purpose of this study was two-fold. First, the motivation for consuming caffeine was investigated among college students. Second, the relationship between the consumption of caffeine and the quality of sleep was evaluated. 

## 2. Materials and Methods

### 2.1. Participants 

Quantitative methods were used in this study to collect data from the respondents. Before conducting the survey, the study was screened and approved by the institutional review board (1041549-191011-SB-80) at Woosong University. A survey was administered to college students in Korea for about four weeks. The survey was conducted on campus and was given to students enrolled at the University of Daejeon in Korea. The purpose of the survey was explained to the study participants and the survey proceeded after the respondents provided consent. The questionnaire was self-administered in a paper-based form and completed voluntarily without incentives. A total of 420 questionnaires were distributed to students and 404 were returned. Respondents were asked if they had consumed caffeine within the last month and 12 who had not consumed caffeine during this period were excluded. After screening, another 11 students were excluded due to incomplete responses and the remaining 381 were included in further analyses. 

### 2.2. Measures

The participants were asked about their motivations for consuming caffeinated beverages and the questions were adapted from previous studies [30,31]. The questions were presented with a 5-point Likert scale (1: *never* ~ 5: *always*). The consumption frequency and the most consumed caffeinated beverages were evaluated. The categories for caffeinated beverage were coffee, tea, energy drinks, and soft drinks and others (chocolate/cocoa drinks), which were taken from a previous study [24] [5,29]. In addition, the times when caffeinated beverages were usually consumed were evaluated. Along with caffeinated beverage consumption behaviors, the sleep quality of the respondents was investigated. In order to measure the quality of sleep, questions from the Pittsburgh Sleep Quality Index (PSQI) [32] were used. Questions such as their usual bed time, number of minutes until they fell asleep, usual wake up time, and hours of actual sleep were asked. Other questions were asked with four scales (not during the past month, less than once a week, once or twice a week, three or more times a week) such as during the past month, (1) how often have you had trouble sleeping because you…; (2) cannot get to sleep within 30 min; (3) wake up in the middle of the night or early morning, etc. All questions were excerpted from a previous study [33]. Lastly, the demographic information of the respondents was collected. 

### 2.3. Data Analysis 

Data were coded using Excel and analyzed with the Statistical Package for the Social Sciences (SPSS, version 25, IBM Corp, 2018). An exploratory factor analysis (EFA) using the maximum likelihood method with the varimax rotation was conducted to identify the factor structure of 36 items related to the motivations for caffeinated beverage consumption by the respondents. The correlations of the extracted factors and their Cronbach’s alpha were determined to evaluate the validity and internal consistency. Descriptive analyses were conducted to investigate the caffeinated beverage consumption behaviors of the students. In addition, analysis of variance (ANOVA) and Tukey’s test were used to compare each factor in the demographic characteristics.

Multiple regression analysis was performed on each category of caffeinated beverages (coffee, tea, energy drinks, soft drinks and others) in order to determine the effectiveness of the motivation factors. All categories of caffeinated beverages were dependent variables and the motivation factors were independent variables. Multiple regression analysis was performed simultaneously for all categories. The frequency of caffeinated beverage consumption was calculated as the sum of the consumption frequencies for all four categories of caffeinated beverage and divided into two groups based on the mean value. An independent *t*-test was conducted on the sleep quality of the low and high-caffeinated beverage consumption groups using PSQI. Lastly, descriptive analysis was performed on the demographic characteristics of the college students.

## 3. Results 

### 3.1. Demographic and Caffeinated Beverage Consumption Characteristics 

Table 1 presents the demographic characteristics of the respondents. One hundred and seventy-two of the respondents were men (45.6%) and 205 were women (54.4%). All respondents were single. About 35.8% of the respondents were freshmen and 30.8% were sophomores. The majority of the respondents were 20 years old (30.2%) and 21 years old (21.2%). More than two-thirds of the respondent reported that they lived alone (71.4%). The majority of the respondents did not smoke (79.3%) and 76.7% of those who smoked were male. In comparisons of caffeinated beverage consumption by gender, only *coffee* consumption showed a significant difference. Male students (Mean [M] = 1, standard deviation [SD] = 0.872) consumed more coffee than female students (M = 0.81, SD = 0.785, t = 2.229, *p* < 0.05). Tea, energy drinks, and soft drinks and others did not show any differences by gender. The consumptions of the four caffeinated beverages were compared according to smoking status. Coffee (t = 3.604, *p* < 0.001) and tea consumption (t = −2.897, *p* < 0.01) significantly differed by smoking status. Smokers (M = 1.22, SD = 0.815) had higher coffee consumption than non-smokers (M = 0.83, SD = 0.831). The opposite pattern was observed for tea consumption, with non-smokers (M = 0.5, SD = 0.809) consuming more tea than smokers (M = 0.26, SD = 0.598). The consumption of energy drinks and soft drinks showed insignificant differences between smokers and non-smokers.

Caffeinated beverage consumption behaviors are shown in Table 2. The majority of the respondents consumed coffee (45.4%), followed by soft drinks and others (26.49%). The consumption of tea (7.79%) and energy drinks (6.5%) was relatively low. Most respondents consumed *coffee* once a day (46.7%) and a smaller proportion consumed coffee twice a day (14.4%). One hundred and thirty-one (34.4%) respondents reported not drinking coffee every day. For *tea*, 25.2% of the respondents reported consumption once a day and 66.7% did not consume tea. For *energy drinks*, the majority of respondents did not consume the beverages every day (88.5%) and 8.7% consumed energy drinks once a day. Similar to coffee, the majority of respondents consumed soft drinks and others once a day (43.3%) followed by two times a day (12.9%). 

The times when caffeinated beverages were consumed varied. The highest proportions of respondents consumed caffeinated beverages between 14:00 and 16:00 (24.4%), followed by between 12:00 and 14:00 (20.1%). Other time periods for consuming caffeinated beverages were almost equally distributed between 16:00–18:00 (13.2%), 18:00–20:00 (14.8%), and after 20:00 (16.6%). The lowest proportion of respondents consumed caffeinated beverages between 07:00-10:00 (6.3%), followed by between 10:00–12:00 (4.7%).

### 3.2. Reliability and Validity of the Measurements 

Exploratory factor analysis was conducted for the 36 items adopted from previous studies [30,31] (Table 3). Six factors were extracted and both the variances and Eigen values for the factors showed satisfactory reliability (Table 4). The six factors were as follows: F1: sensory aspects (α = 0.888), F2: alertness (α = 0.882), F3: social aspects (α = 0.904), F4: health benefit (α = 0.843), F5: mood (α = 0.912), and F6: habit (α = 0.853). For the habit factor, excluding Question 30 increased the reliability from 0.782 to 0.853, hence the question was omitted. Correlations among the six factors were examined and indicated satisfactory validity (Table 5). All factors were significant at the 0.01 level. 

### 3.3. Motivations Influencing Caffeinated Beverage Consumption 

Multiple regression analysis was conducted to determine the effectiveness of motivations for caffeinated beverage consumption. Six motivations were regressed on the frequencies of the four categories of caffeinated beverage (coffee, tea, energy drinks, soft drinks and others). *Coffee* consumption was affected by a desire for alertness (B = 0.107, SE = 0.049, t = 2.181, *p* < 0.05) and habit (B = 0.345, SE = 0.046, t = 7.428, *p* < 0.001) (Table 6). *Tea* consumption was influenced by social motivations (B = 0.142, SE = 0.060, t = 2.357, *p* < 0.05) (Table 7). Both a desire for alertness (B = 0.100, SE = 0.034, t = 2.966, *p* < 0.01) and health benefits (B = 0.120, SE = 0.051, t = 2.345, *p* < 0.05) significantly impacted *energy drink* consumption (Table 8). *Soft drinks and others* were not significantly influenced by any motivations (Table 9). 

The motivations for caffeinated beverage consumption were compared according to the demographic characteristics of the respondents. Independent *t*-tests showed the six motivations were not significantly different by gender. ANOVA and Tukey’s test were conducted to identify differences among the effect of years of college on the motivations. Alertness as a motivation showed significant differences among students (F = 4.223, *p* < 0.01). Seniors (M = 2.862, SD = 1.132) were more likely to consume caffeinated beverage for alertness than freshmen (M = 2.216, SD = 0.949) and sophomores (M = 2.181, SD = 0.931). Only habit as a motivation was significantly different among respondents with different years of college (F = 4.760, *p* < 0.01). Habit as a motivation for freshmen (M = 2.231, SD = 1.112) was significantly different from that for juniors (M = 2.677, SD = 1.169) and seniors (M = 2.949, SD = 1.501), with *p* = 0.05. Independent *t*-tests were conducted on comparisons of motivations according to the smoking status. Sensory aspects (t = 1.982, *p* = 0.05), health benefits (t = 2.089, *p* < 0.05), and habit (t = 2.426, *p* < 0.05) showed significant differences. Sensory aspects (M = 3.00, SD = 1.032), health benefits (M = 1.798, SD = 0.08), and habit (M = 2.748, SD = 0.131) were greater motivations for smokers than for non-smokers (sensory: M = 2.734, SD = 0.059, health benefits: M = 1.610, SD = 1.001, habit: M = 2.387, SD = 1.171). 

### 3.4. Quality of Sleep and Caffeinated Beverage Consumption

The sleep quality of the respondents was measured using PSQI. The average number of hours the students spent sleeping was more than 6 (M = 6.6, SD = 1.640) and the average length of time they spent in bed before falling asleep was 23.65 min (SD = 20.166). The PSQI score was calculated as described in a previous study [32]. The average PSQI score was 5.86 (SD = 3.064), ranging from 0 to 17. The sleep quality had no differences by gender or grade but was affected by smoking (t = 2.621, *p* = 0.01). ANOVA showed that non-smokers had a lower PSQI (M = 5.63, SE = 2.88) than smokers (M = 6.83, SE = 3.67). In addition, the correlation between the consumption of caffeinated beverages and PSQI scores was significant (Pearson’s correlation = 0.126, *p* < 0.05). 

## 4. Discussion

The results of this study indicated that the motivations for caffeinated beverage consumption were sensory effects, alertness, social factors, health benefits, mood, and habit, similar to the results of a previous study [30]. Interestingly, the motivations for the consumption of each caffeinated beverage were different. Coffee drinkers were motivated by a desire for alertness, which is one of the appealing characteristics of caffeine, and drinking coffee was considered a habitual behavior. Tea consumption was motivated by socialization. In this study cohort, tea was less popular than coffee and soft drinks. Tea may not appeal to college students sufficiently for them to be motivated by its caffeine content; however, socializing with others motivated college students to consume tea. This result suggests that socialization may require a compromise regarding the selection of beverages since tea brewed in a pot can serve a group of people with the adjustment of the tea leaves. It has been reported that energy drinks are consumed for various reasons [10]. This study found that college students chose energy drinks due to their well-known reputation of inducing alertness and health benefits. Previous results suggested that college students expect strong caffeine effects from energy drinks and consume them as health aids for digestion, blood pressure, headache, etc. [7]. It may be that college students consider energy drinks as an effective dose of caffeine that can provide health benefits; for example, an increased heart rate improves blood circulation, which may help dieters lose weight [33]. However, it appears that caffeinated beverage consumers do not know or misjudge the adverse effects of caffeine in energy drinks. In order to inform college students about how caffeine works in the human body and the associated mechanisms, appropriate educational intervention should be implemented [10]. Soft drinks and others were more popular than energy drinks and tea but less popular than coffee; however, there were no motivations for consuming soft drinks and others. This can be interpreted to mean that college students do not associate soft drinks with caffeine, possibly due to a lack of awareness of the features of caffeine and its products [10]. College students consume caffeinated beverage products with different motivations, but they appear to be more aware of the positive effects of caffeine than the negative effects. As mentioned earlier, appropriate and effective educational intervention can help highlight both the positive and negative aspects of caffeine consumption, which will help students make smart choices about caffeinated beverages. Effective educational intervention should be developed taking into account the idiosyncratic characteristics of caffeinated beverage consumption [34]. More detailed interventions will facilitate healthier caffeinated beverage consumption behavior. With such educational invention, labels on products are necessary to inform consumers about the amount of caffeine in each product, similar to nutritional fact labeling. This will help consumers be more informed about caffeine in the products. 

Smokers had a higher intake of coffee than non-smokers, who had a higher intake of tea; however, no differences were found in the intake of energy drinks and soft drinks unlike in a previous study [11]. These results suggest that smokers may prefer coffee when they smoke. Smokers might enjoy the full power of the enhanced caffeine effect physically or emotionally. Hence, physiological, cognitive, and environmental factors may all contribute to the association between smoking and caffeine intake [11]. Labels cautioning users that coffee consumption while smoking can increase the effect of caffeine may be beneficial to educate and warn consumers not to abuse cigarettes and coffee.

Measuring the sleep quality of college students using PSQI showed that they had some difficulties sleeping. PSQI scores > 5 indicate the individual is having some difficulties sleeping, representing poor sleep quality [32]. The average sleep duration, which was almost 7 hours, was moderate and the time spent in bed before falling asleep was 23 min, similar to the results from insomniac adults in Korea [35]. Sleep quality may be poor and the caffeinated beverage consumption was correlated with sleep quality, similar to previous studies [18,22,23,36]. However, a study conducted in three European nations showed that caffeine did not cause difficulties in sleep after controlling for some demographics [37]. There may be other factors that affect sleep quality such as electronic device usage, lifestyle, and fast food consumption. Furthermore, sleep quality did not differ by gender unlike previous findings [29]. Interestingly, non-smokers had better sleep quality than smokers though gender and grade showed no differences in sleep quality. Smokers may have different reasons for poor sleep quality regardless of caffeinated beverage consumption. There may also be confounding effects from other elements since this was a self-reported survey and the number of variables examined in the questionnaire was limited, primarily focusing on the motivations for caffeinated beverage consumption and sleep quality. 

Caffeinated beverages are prevalent in daily life and have become a normal part of life. College students consume caffeinated beverage from various sources and coffee was the most common caffeine product consumed by the respondents in this study, followed by soft drinks, tea, and energy drinks, similar to the pattern reported in previous studies [4,11]. In the general population, the frequency of tea intake is higher than that of energy drinks [4]. However, the respondents in this study, college students, had a higher intake of energy drinks than tea. Caffeinated beverage consumption comparisons by gender showed that male students had more coffee intake than female students, similar to a previous report that adult men (over 18 years) consumed more caffeine from beverages than adult women [4]. Adolescents showed a similar pattern [23], but another study found that women consume more caffeinated drinks than men [11]. The results of this study indicated that the majority of smokers were male students and this group is more likely to consume coffee while smoking cigarettes, a correlation extensively reported in the literature [11,34].

## 5. Limitations 

The effects of caffeinated beverages may be better understood in combination with other food-related activities and individual lifestyles [22]. There are some factors that affect sleep other than caffeine. For example, noise, artificial light, and diet might interfere with sleep and these factors should be included in future research. In addition, electronic media usage should be included in a future study.

This study did not measure the amount of caffeine intake. Rather, it focused on the frequency of consumption and motivations related to smoking and sleep quality. The caffeine content of products depends on the method of preparation, brand, and serving size; hence, variations exist [1]. Calculating the amount of caffeine in products would be unreliable in a self-reported survey; therefore, this study did not assess the actual caffeine amount. Future observation or laboratory studies to examine the retail caffeinated beverage products available in Korea may be needed. Specific cases of caffeinated beverage consumption, for example, personal, physical, and societal environmental factors should be investigated to assess consumer behaviors regarding caffeine. In addition, this study was conducted on college students in Korea and the generalization of the results may be limited. 

## 6. Conclusions

The purpose of this study was to conduct specific analyses of the behavioral characteristics of caffeine consumption in accordance with motivation. Several distinct features were identified in terms of caffeinated beverage motivation and caffeinated beverage consumption. Respondents had poor sleep quality, and relationships between caffeinated beverage consumption and sleep quality were found using PSQI. Smokers had higher coffee intake and lower tea consumption than non-smokers. In addition, smokers had poor sleep quality. Interactions between caffeinated beverage consumption and smoking had no effect on sleep quality. College students appeared to be poorly informed about the characteristics of caffeine; therefore, appropriate educational intervention should be implemented. Providing labeling information about caffeine content and the daily limit may be a good educational tool for consumers. Moreover, tailoring detailed information for specific cases such as the fact that consuming coffee while smoking increases the effect of caffeine should be applied as an educational tool and labeling policy.

## Figures and Tables

**Table 1:** Demographic characteristics and caffeinated beverage consumption behaviors (*n* = 381).

Characteristics		Frequency (%)	Caffeine Consumption *	Coffee	Tea	Energy Drinks	Soft Drinks and Others
			**Mean** ± **Standard Deviation**				
Gender	Male	172 (45.6)	2.331 ± 1.552	1.00 ± 0.872 ^a^	0.39 ± 0.745	0.18 ± 0.429	0.76 ± 0.828
	Female	205 (54.4)	2.312 ± 1.572	0.81 ± 0.785 ^b^	0.53 ± 0.820	0.11 ± 0.434	0.860 ± 0.858
	Missing	4					
Marital status	Married	0 (0)					
	Single	377 (100)	2.321 ± 1.561	0.90 ± 0.830	0.46 ± 0.788	0.14 ± 0.432	0.82 ± 0.845
	Missing	4					
Grade	Freshman	135 (35.8)	2.289 ± 1.450	0.84 ± 0.775	0.42 ± 0.738	0.16 ± 0.403	0.87 ± 0.796
	Sophomore	116 (30.8)	2.285 ± 1.651	0.81 ± 0.790	0.52 ± 0.807	0.11 ± 0.412	0.84 ± 0.881
	Junior	100 (26.5)	2.420 ± 1.659	1.02 ± 0.899	0.43 ± 0.856	0.15 ± 0.479	0.82 ± 0.903
	Senior	26 (6.9)	2.270 ± 1.373	1.12 ± 0.952	0.58 ± 0.703	0.19 ± 0.491	0.38 ± 0.571
	Missing	4					
Age	20 years old	114 (30.2)	2.167 ± 1.310	0.79 ± 0.722	0.41 ± 0.714	0.15 ± 0.382	0.82 ± 0.736
	21	80 (21.2)	2.388 ± 1.555	0.78 ± 0.779	0.55 ± 0.855	0.09 ± 0.363	0.93 ± 0.104
	22	51 (13.5)	2.608 ± 2.050	1.10 ± 0.922	0.43 ± 0.700	0.14 ± 0.530	1.03 ± 0.144
	23	51 (13.5)	2.177 ± 1.600	0.80 ± 0.825	0.43 ± 0.831	0.20 ± 0.530	0.74 ± 0.104
	24	41 (10.9)	2.317 ± 1.312	1.10 ± 0.768	0.46 ± 0.840	0.22 ± 0.475	0.55 ± 0.086
	25	26 (6.9)	2.462 ± 1.816	1.19 ± 1.132	0.42 ± 0.920	0.12 ± 0.431	1.12 ± 0.219
	26 and above	14 (3.7)	2.429 ± 1.604	0.93 ± 0.830	0.71 ± 0.825	0.07 ± 0.267	0.83 ± 0.221
	Missing	4					
Place of residence	Live in board and lodging	4 (1.1)	2.000 ± 1.414	0.25 ± 0.500	0.00 ± 0.00	0.25 ± 0.500	1.50 ± 1.291
	Live alone	267 (71.4)	2.296 ± 1.504	0.95 ± 0.828	0.42 ± 0.748	0.15 ± 0.439	0.78 ± 0.812
	School dormitory	16 (4.3)	2.875 ± 2.825	1.06 ± 1.063	0.69 ± 0.946	0.19 ± 0.544	0.94 ± 1.063
	Live with parents	87 (23.3)	2.310 ± 1.458	0.74 ± 0.784	0.60 ± 0.882	0.13 ± 0.398	0.85 ± 0.870
	Missing	7					
Do you smoke?	Yes	74 (20.7)	2.432 ± 1.745	1.22 ± 0.815 ^a^	0.26 ± 0.598 ^a^	0.20 ± 0.496	0.76 ± 0.948
	No	283 (79.3)	2.307 ±1.507	0.83 ±0.831 ^b^	0.50 ± 0.809 ^b^	0.14 ± 0.436	0.84 ± 0.825
	Missing	24					

^a,b^ indicate significant differences in column for the category. * caffeine was consumed more than twice daily by respondents.

**Table 2:** Characteristics of the respondents regarding their caffeinated beverage consumption.

Caffeinated Beverage Consumption Behaviors		Frequency	Valid Percentage (%)
Major caffeinated beverages consumed a day (multiple answers)	Coffee	175	45.4
	Tea	30	7.79
	Energy drinks	25	6.5
	Coffee and tea	53	13.77
	Soft drinks and others	102	26.49
Average coffee consumption a day	0	131	34.4
	1	178	46.7
	2	55	14.4
	3	13	3.4
	4 and above	4	1.0
Average tea consumption a day	0	254	66.7
	1	96	25.2
	2	19	5.0
	3	7	1.8
	4 and above	5	1.3
Average energy drinks consumption a day	0	337	88.5
	1	33	8.7
	2	10	2.6
	3	1	0.3
	4 and above	0	0
Average soft drinks and others consumption a day	0	152	39.9
	1	165	43.3
	2	49	12.9
	3	10	2.6
	4 and above	5	1.3
Major times for consuming caffeinated beverages (multiple answers)	07–10	28	6.3
	10–12	21	4.7
	12–14	90	20.1
	14–16	109	24.4
	16–18	59	13.2
	18–20	66	14.8
	After 20	74	16.6

**Table 3:** Measurement items for motives for caffeine consumption questionnaire.

Measurement Items *	Strongly Disagreed	Disagreed	Neutral	Agreed	Strongly Agreed
	Valid %				
Q1 It has become a ritual for me.	27.0	29.1	21.0	14.2	8.7
Q2 It helps me when I am tired.	13.1	22.0	18.4	29.9	16.5
Q3 I like its taste.	11.8	15.7	24.1	31.8	16.5
Q4 It helps me to concentrate.	22.9	22.1	24.7	21.6	8.7
Q5 It reduces headaches.	65.6	17.5	12.4	2.9	1.6
Q6 It improves my mood.	36.1	22.7	19.3	15.8	6.1
Q7 Everyone on campus drinks it.	68.4	19.2	7.4	4.2	.8
Q8 It is a pleasant ritual.	36.1	20.8	21.8	13.7	.6
Q9 It peps me up.	29.2	22.4	26.1	15.8	6.6
Q10 Drinking caffeinated beverages is important in social situations.	36.1	26.3	19.5	14.5	3.7
Q11 It helps me to stay awake.	21.6	18.2	19.5	28.2	12.6
Q12 My mood becomes better.	33.3	24.6	21.2	15.6	5.3
Q13 I become refreshed.	34.7	24.5	23.2	12.9	4.7
Q14 Drinking caffeinated beverages is a social event.	37.5	28.5	19.0	12.9	2.1
Q15 It adds good atmosphere to conversation.	28.3	24.1	26.8	16.3	4.5
Q16 It helps me to wake up.	14.7	18.2	21.1	27.6	18.4
Q17 It is delicious.	11.3	16.1	22.4	32.2	17.9
Q18 It stimulates me.	29.9	24.9	24.3	14.6	6.3
Q19 It brings me together with other people.	30.2	25.1	25.7	14.3	4.8
Q20 It invigorates me.	23.0	23.8	26.5	18.8	7.9
Q21 It feels good during a conversation.	36.7	19.3	27.7	14.5	1.8
Q22 I feel physically and mentally fitter.	48.5	24.0	17.7	8.2	1.6
Q23 It is good for my blood pressure.	67.5	19.3	9.8	2.9	.5
Q24 I love the smell.	16.6	17.9	25.1	23.7	16.6
Q25 It has become part of my everyday life.	36.4	19.0	16.1	16.9	11.6
Q26 It makes me more motivated to work.	28.8	24.3	21.9	17.4	7.7
Q27 It relieves tension.	37.0	24.5	23.7	11.7	3.2
Q28 It calms me down.	36.9	23.7	23.7	11.3	4.2
Q29 It is good to relax with a cup of a caffeinated beverage.	30.6	15.0	26.4	21.1	6.9
Q30 It feels good when I am smoking a cigarette.	78.6	7.4	6.9	4.5	2.6
Q31 It helps me digest.	57.6	21.3	13.7	5.5	1.8
Q32 It hydrates me.	52.8	19.5	16.4	9.5	1.8
Q33 Sometimes it feels good to be stimulated.	36.3	22.6	26.6	11.1	3.4
Q34 It makes me feel that I am full of energy.	28.6	20.6	28.8	17.7	4.2
Q35 I love the atmosphere associated with it.	29.7	22.9	25.3	15.8	6.3

* 5-point Likert scales (1: strongly disagreed ~ 5: strongly agreed).

**Table 4:** Exploratory factor analysis of the motivations for consuming caffeinated beverages (*n* = 381).

Measurement Items *	Sensory	Alertness	Social	Energize	Mood	Habit
Q3 I like the taste.	0.707					
Q17 It is delicious.	0.709					
Q24 I love the smell.	0.753					
Q27 It relieves tension.	0.583					
Q28 It calms me down.	0.588					
Q29 It is good to relax with a cup of a caffeinated beverage.	0.674					
Q2 It helps me when I am tired.		0.833				
Q4 It helps me to concentrate.		0.666				
Q11 It helps me to stay awake.		0.824				
Q16 It helps me to wake up.		0.891				
Q18 It stimulates me.		0.401				
Q20 It invigorates me.		0.527				
Q26 It makes me more motivated to work.		0.501				
Q10 Drinking caffeinated beverages is important in social situations.			0.654			
Q14 Drinking caffeinated beverages is a social event.			0.820			
Q15 It adds a good atmosphere to conversation.			0.789			
Q19 It brings me together with other people.			0.836			
Q21 It feels good during a conversation.			0.669			
Q35 I love the atmosphere associated with it.			0.503			
Q5 It reduces headaches.				0.620		
Q7 Everyone on campus drinks it.				0.432		
Q22 I feel physically and mentally fitter.				0.604		
Q23 It is good for my blood pressure.				0.648		
Q31 It helps me digest.				0.597		
Q32 It hydrates me.				0.509		
Q33 Sometimes it feels good to be stimulated.				0.721		
Q6 It improves my mood.					0.712	
Q9 It peps me up.					0.575	
Q12 My mood becomes better.					0.678	
Q13 I become refreshed.					0.635	
Q33 Sometimes it feels good to be stimulated.					0.465	
Q34 It makes me feel that I am full of energy.					0.565	
Q1 It has become a ritual for me.						0.593
Q8 It is a pleasant ritual.						0.489
Q25 It has become part of my everyday life.						0.544
Q30 It feels good when I am smoking a cigarette.						0.543
Eigen value	4.806	4.452	4.439	4.352	4.313	1.982

* 5-point Likert scales (1: strongly disagreed ~ 5: strongly agreed).

**Table 5:** Correlations between the factors and internal consistency.

	Sensory	Alertness	Social	Energize	Mood	Habit
Sensory						
Alertness	0.507 **					
Social	0.605 **	0.476 **				
Health benefits	0.600 **	0.440 **	0.604 **			
Mood	0.725 **	0.691 **	0.636 **	0.650 **		
Habit	0.689 **	0.567 **	0.595 **	0.546 **	0.696 **	
Cronbach’s alphas	0.888	0.882	0.904	0.843	0.912	0.853

** Significant correlations at 0.01 level.

**Table 6:** Results of regression analysis of motivations influencing *coffee* consumption.

Model	Unstandardized			
	B	SE	St.d Beta	t-Value
Constant	−0.463	0.122		−3.793 ***
Sensory	0.108	0.055	0.133	1.944
Alertness	0.107	0.049	0.129	2.181 *
Social	0.023	0.052	0.027	0.444
Energize	0.011	0.075	0.009	0.148
Mood	−0.072	0.066	−0.088	−1.093
Habit	0.345	0.046	0.489	7.428 ***

F = 36.394, *p* = 0.000, R = 0.626, R^2^ = 0.392, Adjusted R^2^ = 0.382. * *p* < 0.05, *** *p* < 0.001.

**Table 7:** Results of regression analysis of motivations influencing *tea* consumption.

Model	Unstandardized			
	B	SE	St.d Beta	t-Value
Constant	0.309	0.143		2.162
Sensory	0.048	0.065	0.064	0.744
Alertness	−0.056	0.058	−0.073	−0.979
Social	0.142	0.060	0.180	2.357 *
Energize	0.098	0.088	0.086	1.114
Mood	−0.115	0.077	−0.152	−1.490
Habit	−0.025	0.054	−0.039	−0.467

F = 2.070, *p* = 0.560, R = 0.188, R^2^ = 0.035, Adjusted R^2^ = 0.018. * *p* < 0.05.

**Table 8:** Results of regression analysis of motivations that influence *energy drink* consumption.

Model	Unstandardized			
	B	SE	St.d Beta	t-Value
Constant	0.045	0.083		0.534
Sensory	−0.050	0.038	−0.113	−1.330
Alertness	0.100	0.034	0.219	2.966 **
Social	−0.006	0.035	−0.013	−0.176
Health benefits	0.120	0.051	0.180	2.345 *
Mood	−0.042	0.045	−0.094	−0.931
Habit	−0.046	0.032	−0.120	−1.453

F = 2.925, *p* = 0.009, R = 0.222, R^2^ = 0.049, Adjusted R^2^ = 0.032. * *p* < 0.05, ** *p* < 0.01.

**Table 9:** Results of regression analysis of motivations that influence the consumption of *soft drinks and others*.

Model	Unstandardized			
	B	SE	St.d Beta	t-Value
Constant	0.889	0.162		5.473 ***
Sensory	−0.067	0.074	−0.078	−0.904
Alertness	−0.068	0.065	−0.078	−1.039
Social	0.064	0.069	0.072	0.937
Health benefits	0.151	0.100	0.118	1.516
Mood	0.032	0.088	0.038	0.368
Habit	−0.060	0.062	−0.081	−0.966

F = 1.111, *p* > 0.05, R = 0.139, R^2^ = 0.019, Adjusted R^2^ = 0.002. *** *p* < 0.001.
